# Comparative Efficacy and Safety of Antibody–Drug Conjugates Versus Standard Chemotherapy in Metastatic Breast Cancer: A Comprehensive Systematic Review and Meta-Analysis

**DOI:** 10.7759/cureus.98924

**Published:** 2025-12-10

**Authors:** Hagar Teama, Ahmed Elnewishy, Mahmoud Noureldin, Omar Elnewishy

**Affiliations:** 1 Clinical Pharmacy, Faculty of Pharmacy, Kafr El Sheikh University, Kafr El Sheikh, EGY; 2 Trauma and Orthopaedics, Maidstone and Tunbridge Wells NHS Trust, Royal Tunbridge Wells, GBR; 3 Trauma and Orthopaedics, University Hospitals Sussex NHS Foundation Trust, Brighton, GBR; 4 Medicine, Cairo University, Cairo, EGY

**Keywords:** antibody-drug conjugates, chemotherapy, drug efficacy, her2-positive breast cancer, metastatic breast cancer, objective response rate, overall survival, progression-free survival, safety, triple negative breast cancer

## Abstract

Antibody-drug conjugates (ADCs) represent a novel class of therapeutics that link monoclonal antibodies to potent cytotoxic drugs, enabling targeted delivery and reduced systemic toxicity compared with conventional chemotherapy. In metastatic breast cancer (MBC), ADCs have emerged as an important treatment option across human epidermal growth factor receptor 2 (HER2)-positive, HER2-low, and triple-negative subtypes. This systematic review and meta-analysis evaluated the efficacy and safety of ADCs compared with standard chemotherapy by analyzing data from nine randomized controlled trials including 3,498 patients. The pooled results demonstrated that ADCs significantly improved overall survival (OS) and objective response rates while maintaining comparable progression-free survival and showing no increase in grade 3 or higher adverse events. Heterogeneity was low for OS but higher for response rates, reflecting variability among drug types and patient populations. Safety analyses showed that ADCs were generally well tolerated, with fewer severe hematologic and gastrointestinal toxicities than chemotherapy, although specific toxicities such as interstitial lung disease with trastuzumab deruxtecan require ongoing monitoring. Overall, the evidence confirms that ADCs provide superior therapeutic benefits and better tolerability than conventional chemotherapy in MBC, marking a significant advance toward more precise and effective systemic treatment strategies that improve survival and quality of life for patients with advanced disease.

## Introduction and background

Breast cancer accounted for 2.26 million new cases in 2020 [1], with the highest incidence in high-income regions such as North America and Western Europe, rapidly rising rates in parts of Asia, South America, and North Africa, and a persistently high contribution to cancer mortality worldwide. Up to 30% of patients initially diagnosed with early-stage disease develop incurable metastatic breast cancer (MBC), necessitating lifelong treatment [2]. Survival for MBC varies by region, development level, and subtype; the global five-year survival for all breast cancer is ~73% [3], while the 10-year survival for MBC in the Netherlands is 9% [4]. Circulating tumor DNA and circulating tumor cells show promise for predicting response and overall survival (OS), enabling more personalized monitoring strategies [5].

Despite advances in targeted and hormonal therapies, chemotherapy remains integral to MBC care, commonly used as single agents or in combinations to treat progression or provide palliation, with ongoing efforts to optimize sequencing and minimize toxicity [6]. In metastatic metaplastic breast cancer, palliative chemotherapy was associated with improved median OS (8 to 12 months) compared with no chemotherapy [7]. Chemotherapy also retains a key role in resistant states, such as hormone receptor-positive MBC after endocrine progression, where clinical benefit occurs regardless of prior endocrine response [8] and in HER2-amplified, hormone receptor-positive disease, where multiple lines may prolong treatment duration and improve survival [9]. However, chemotherapy is limited by high toxicity, nonselective tumor targeting, and diminishing efficacy with successive lines; median progression-free survival (PFS) typically decreases from ~5 months in first line to ~3 months by third or fourth line [10], and systemic adverse effects (e.g., neutropenia, neuropathy, gastrointestinal symptoms) substantially impact quality of life [11].

Antibody-drug conjugates (ADCs) aim to reduce systemic toxicity in MBC, and a meta-analysis of seven randomized trials reported better PFS and OS versus chemotherapy across human epidermal growth factor receptor 2 (HER2)-positive, HER2-low, and triple-negative subtypes, with favorable hazard ratios [12]. Beyond prolonging survival, ADCs broaden eligibility to patients with low biomarker expression; trastuzumab deruxtecan (T-DXd) exerts a bystander effect with activity irrespective of HER2 status [13], and real-world data on sequential ADC use suggest benefits in heavily pretreated populations, with signals for improved outcomes when used earlier [14]. ADCs have emerged as a new therapeutic class in MBC, with United States Food and Drug Administration approvals for ado-trastuzumab emtansine (T-DM1), T-DXd, and sacituzumab govitecan (SG) following randomized trials demonstrating superiority over standard options in HER2-positive and triple-negative disease [15]; T-DXd and SG have changed practice by offering options for HER2-low tumors and for heavily pretreated patients [16]. Phase 3 studies leading to regulatory approvals (e.g., DESTINY-Breast03, ASCENT, TROPION-Breast01) showed significant PFS and OS gains versus chemotherapy in relevant subgroups, shifting clinical guidelines [17]. A pooled analysis of seven randomized trials (>5,000 patients) confirmed ADC-associated improvements in PFS and OS over chemotherapy in HER2-positive and triple-negative MBC, and in DESTINY-Breast04, patients with HER2-low disease treated with T-DXd had a 47% reduction in mortality risk versus chemotherapy [18].

Review objective

This systematic review and meta-analysis evaluate the efficacy and safety of ADCs compared with standard chemotherapy in MBC.

Methods

The review question was structured using the PICO (Population, Intervention, Comparator, and Outcome) framework: adult patients with metastatic or unresectable breast cancer (Population) treated with antibody drug conjugates (Intervention) compared with standard single agent chemotherapy or active control regimens (Comparison), with outcomes including PFS, OS, objective response rate (ORR), and grade ≥ 3 adverse events (AEs) (Outcomes).

Search Strategy

In October 2025, we searched PubMed, Scopus, Google Scholar, and the Cochrane Central Register of Controlled Trials (CENTRAL) using a combination of Medical Subject Headings and free text terms. For PubMed, the core strategy combined Boolean operators as follows: (“antibody drug conjugate” OR “ADC” OR “trastuzumab deruxtecan” OR “trastuzumab emtansine” OR “sacituzumab govitecan”) AND (“metastatic breast cancer” OR “advanced breast cancer”) AND (randomized OR randomised OR “phase III”). Similar logic-based combinations were adapted for Scopus, CENTRAL, and Google Scholar. For each database, we screened all records returned on the search date, and for Google Scholar, we reviewed the first 20 pages of results. Reference lists of included articles and relevant reviews were also screened to identify additional eligible trials.

Inclusion Criteria

Randomized controlled trials were eligible if they enrolled adults (≥18 years) with metastatic or unresectable breast cancer, compared an antibody drug conjugate (trastuzumab deruxtecan, trastuzumab emtansine, sacituzumab govitecan, or datopotamab deruxtecan) with standard single-agent chemotherapy or an active control regimen, and reported at least one key clinical outcome. Eligible outcomes included progression-free survival or OS, ORR as defined by RECIST, or grade ≥ 3 treatment-related AEs according to CTCAE. Only full-text articles published in English with extractable quantitative data were included.

Exclusion Criteria

We excluded non-randomized or single-arm studies, trials that did not include a direct comparison between an ADC and standard chemotherapy, and studies that did not report extractable data for any of the prespecified outcomes. Reports with overlapping patient populations were handled by including the most complete or recent dataset and excluding companion analyses. We also excluded conference abstracts, narrative reviews, editorials, commentaries, and non-primary research because these did not provide sufficient detail or quantitative data for reliable synthesis.

Outcome Measures

Outcomes included PFS and OS, ORR (complete or partial response per RECIST), and grade ≥3 AEs (severe or life-threatening per CTCAE), enabling comprehensive efficacy and safety comparisons of ADCs versus chemotherapy [12,19].

Data Extraction and Quality Assessment

Two reviewers used standardized forms to extract study design, sample size, demographics, treatment protocols, follow-up, and outcomes; disagreements were resolved by consensus or third-party adjudication. Risk of bias was assessed with the Cochrane RoB 2 tool [20] across randomization, deviations from intended interventions, missing outcome data, outcome measurement, and selective reporting, with judgments of low risk, some concerns, or high risk.

Statistical Analysis

Analyses were conducted in Review Manager (RevMan) 5.4 (The Cochrane Collaboration, London, UK) [20]. We synthesized odds ratios with 95% confidence intervals for PFS, OS, ORR, and grade ≥3 AEs. Fixed-effect models were used for low heterogeneity (I² <50%); random-effects models were used for moderate to high heterogeneity (I² ≥50%), as assessed by Chi² and I² statistics. Publication bias was evaluated by visual inspection of funnel plots and Egger’s regression (p < 0.05).

The protocol for this review was not prospectively registered in PROSPERO, which we acknowledge as a methodological limitation.

## Review

Results

Search Results and Study Selection

**Figure 1:**
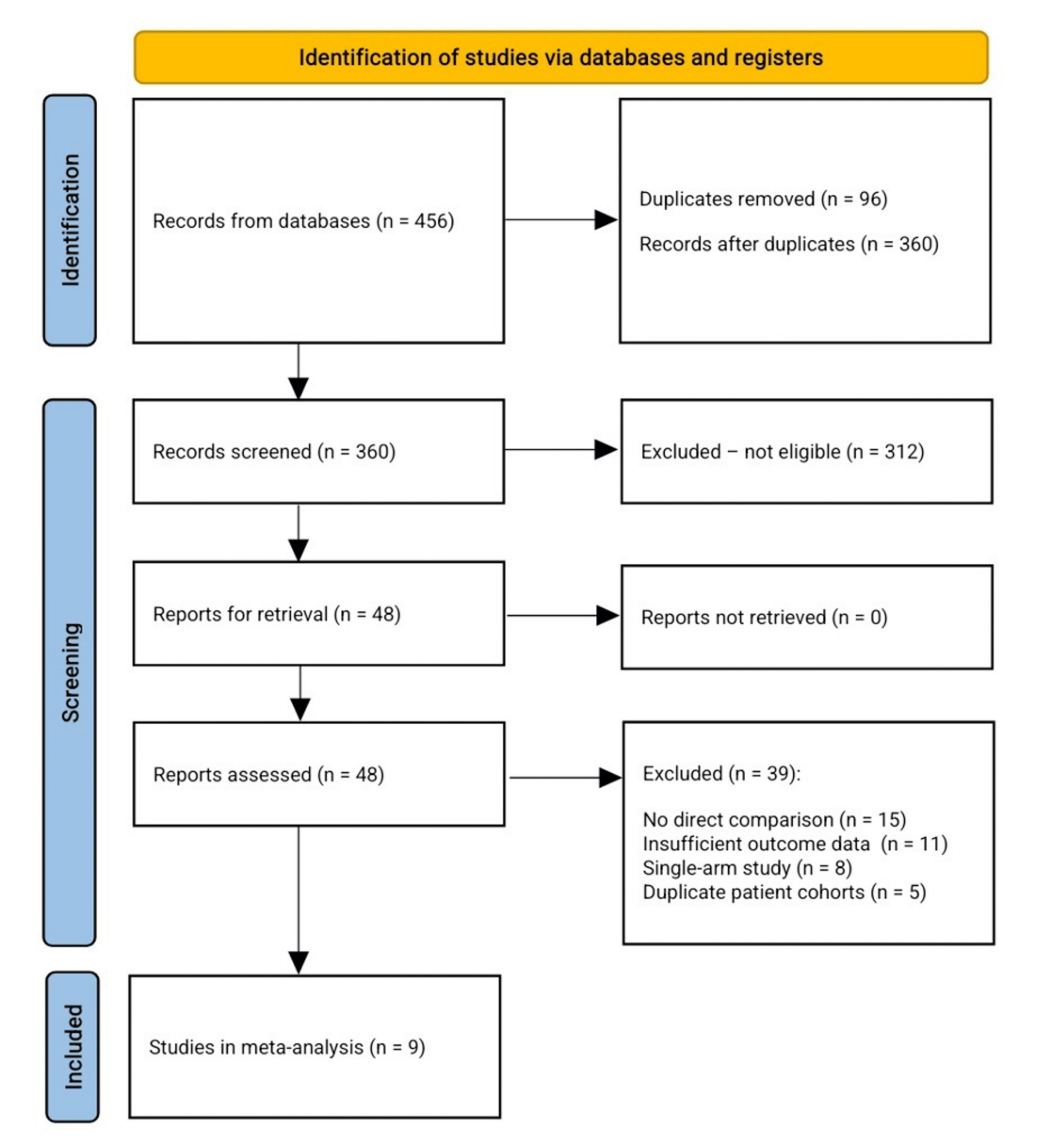
PRISMA flow chart for the included studies PRISMA: Preferred Reporting Items for Systematic Reviews and Meta-Analyses [21].

A total of 456 articles were found in the literature search, with 96 duplicates removed (n = 360), 312 articles excluded during title and abstract screening (n = 48) due to lack of direct comparisons between ADCs and standard chemotherapy in MBC patients, and 48 articles remaining for full-text assessment. Thirty-nine studies were subsequently excluded. Reasons for exclusion comprised: no direct ADC-chemotherapy comparison (n = 15), inadequate outcome data for PFS or OS (n = 11), non-randomized or single-arm designs (n = 8), and patient populations that overlapped with larger trials (n = 5). Nine randomized controlled trials satisfied all inclusion criteria. These trials formed the basis for the meta-analysis examining ADC efficacy and safety against standard chemotherapy in MBC (Figure 1).

Study Characteristics

This systematic review and meta-analysis include nine randomized, open-label, multicenter phase III trials comparing ADCs, trastuzumab deruxtecan (T-DXd), trastuzumab emtansine (T-DM1), sacituzumab govitecan (SG), and datopotamab deruxtecan (Dato-DXd) against standard single-agent chemotherapy or active controls in metastatic/unresectable breast cancer. The aggregated sample comprised 6,186 patients (ADC n=3,579, control n=2,607) across global sites. Populations spanned HER2-positive (first-line and previously treated), HER2-low, HR+/HER2- endocrine-resistant, and triple-negative disease. Cohorts were predominantly female with median age ~53-57 years, ECOG 0-1 in most, visceral disease common, and baseline brain metastases permitted in several trials (typically capped at ≤15%; excluded from the ASCENT primary efficacy set).

Treatment protocols employed standardized three-weekly administration: T-DXd at 5.4 mg/kg at three-week intervals, T-DM1 at 3.6 mg/kg every three weeks, Dato-DXd at 6 mg/kg every three weeks, and SG at 10 mg/kg administered on days 1 and 8 of 21-day cycles. Control arms consisted of physician-selected single-agent chemotherapy (eribulin, vinorelbine, capecitabine, gemcitabine, or taxanes), capecitabine plus lapatinib combination therapy, or trastuzumab combined with taxane. Response assessment utilized RECIST v1.1 criteria. Most trials incorporated blinded independent central review (BICR) for imaging evaluation. PFS served as the primary endpoint in most studies, though Dato-DXd trials designated both PFS and OS as dual primary endpoints. Secondary endpoints encompassed OS, ORR, duration of response (DoR), and patient-reported outcomes when specified in trial protocols. Safety assessments focused on Grade ≥ 3 adverse events (AEs) according to CTCAE criteria, treatment-related discontinuations, and specific toxicities of interest. Interstitial lung disease/pneumonitis monitoring was prioritized for T-DXd and Dato-DXd. SG trials emphasized surveillance for myelosuppression and diarrhea, while T-DM1 studies tracked thrombocytopenia and transaminase elevations. Regular left ventricular ejection fraction (LVEF) assessments were conducted for all HER2-directed antibody-drug conjugates.

Follow-up ranged from ~10-11 months (recent/interim datasets) to ~18-22 months (DESTINY trials) and ~30-48 months in mature OS updates (EMILIA, TH3RESA), enabling robust assessment of early disease control (6- and 12-month PFS landmarks), medians for PFS/OS, and late survival. Collectively, these trials show that modern ADCs consistently improve PFS and frequently extend OS versus chemotherapy across biological subgroups, with distinct but manageable toxicity profiles under proactive surveillance. A study-by-study summary of efficacy and safety is presented in Table 1.

**Table 1:** Key characteristics of the included randomized trials comparing antibody–drug conjugates (ADCs) versus standard single-agent chemotherapy in metastatic breast cancer (MBC) ADC: Antibody–Drug Conjugate; PFS: Progression-Free Survival; OS: Overall Survival; ORR: Objective Response Rate; DoR: Duration of Response; AE: Adverse Event; TRAE: Treatment-Related Adverse Event; HER2: Human Epidermal Growth Factor Receptor 2; HR: Hazard Ratio; CI: Confidence Interval; ECOG: Eastern Cooperative Oncology Group performance status; mTNBC: Metastatic Triple-Negative Breast Cancer.

Category	Study Design	Sample Size (ADC/Chemo)	Level of Evidence	Patient Demographics	Intervention Details	Follow-up Duration	Outcome Measures	Results	Complications	Conclusion
André et al. [22]	Randomized, open-label, global phase III RCT; T-DXd vs TPC; primary PFS (BICR), key secondary OS	406 / 202	Level I	Median age ~54; ~99% female; HR+ ~59% vs 58%; prior pertuzumab ~78% vs 77%; visceral ~78–79%; brain mets 18% both	T-DXd 5.4 mg/kg IV q3w vs TPC (capecitabine+trastuzumab or capecitabine+lapatinib); RECIST v1.1 (BICR); ILD adjudication	Median 21.5 mo (T-DXd) vs 18.6 mo (TPC); DCO Jun 30, 2022	Primary: PFS (BICR). Key secondary: OS. Others: ORR, DoR, investigator PFS, safety (incl. ILD/LVEF)	PFS: 17.8 vs 6.9 mo, HR 0.36; OS: 39.2 vs 26.5 mo, HR 0.66; ORR: 70% vs 29% (CR 14% vs 5%)	Any-grade: nausea 73%, vomiting 38%, alopecia 37%, fatigue 36%; ≥G3: 53% vs 44%; ILD/pneumonitis: 10% (incl. 2 grade-5) vs <1%; median ILD onset ~30 wks	T-DXd markedly improved PFS/OS/ORR vs TPC; ILD risk needs monitoring
Bardia et al. [23]	Randomized, open-label phase III RCT; SG vs single-agent chemo; primary PFS (BICR) in no-brain-mets set	267 / 262 (ITT); 235 / 233 (primary set)	Level I	Median age 54; prior taxane 100%; median prior regimens ~3; ECOG 0–1; visceral common; 61 with brain mets at baseline	SG 10 mg/kg IV D1 & D8 q21d vs eribulin/vinorelbine/capecitabine/gemcitabine; stratified by prior lines, brain mets, region; no crossover	DCO Mar 11, 2020; median FU 17.7 mo	Primary: PFS (BICR, no-brain-mets). Key secondary: OS. Others: inv. PFS, ORR, DoR, safety	No-brain-mets: PFS 5.6 vs 1.7 mo, HR 0.41; OS 12.1 vs 6.7 mo, HR 0.48; ORR 35% vs 5%; DoR 6.3 vs 3.6 mo. ITT: PFS 4.8 vs 1.7 (HR 0.43); OS 11.8 vs 6.9 (HR 0.51)	Any-grade (SG vs chemo): neutropenia 63% vs 43%, diarrhea 59% vs 12%, nausea 57% vs 26%, alopecia 46% vs 16%; ≥G3: neutropenia 51% vs 33%, diarrhea 10% vs <1%, anemia 8% vs 5%, FN 6% vs 2%; discontinuation 5% both; deaths 3 vs 3; low ILD signal	SG improved PFS/OS/ORR with a manageable myelosuppression/diarrhea profile; supports SOC in pretreated mTNBC
Bardia et al. [24]	Randomized, open-label, global phase III RCT; Dato-DXd vs investigator’s-choice chemo; dual primary PFS (BICR) and OS	365 / 367 (ITT); safety 360 / 351	Level I	Median age 56 vs 54; ~99% female; ECOG 0–1; metastatic ~98–100%; visceral common; median ~3 prior lines; prior CDK4/6 ~83%; brain mets ~10% vs 6%	Dato-DXd 6 mg/kg IV q3w vs eribulin/capecitabine/vinorelbine/gemcitabine; antiemetic/oral care guidance; ocular monitoring; exclusions for prior topo-I–ADCs/TROP2	DCO Jul 17, 2023; median FU 10.8 mo; on-treatment 25.8% vs 11.1%	Primary: PFS (BICR), OS. Secondary: inv. PFS, ORR, DoR, DCR (12 wks), TFST/TSST, PFS2, safety	PFS: HR 0.63, P<.0001; medians 6.9 vs 4.9 mo. ORR 36.4% vs 22.9%; DoR 6.7 vs 5.7 mo; DCR 75.3% vs 63.8%; OS HR 0.84 (immature)	Any-grade TRAEs: 93.6% vs 86.3%; ≥G3: 20.8% vs 44.7%. Dato: nausea 51.1% (≥G3 1.4%), stomatitis 50% (≥G3 6.4%), alopecia 36%, fatigue 23.6% (≥G3 1.7%); neutropenia grouped 10.8% (≥G3 1.1%) vs 42.5% (≥G3 30.8%); ILD/pneumonitis 3.3% (mostly G1–2; one G5); ocular surface events mild	Dato-DXd improved PFS with markedly lower high-grade hematologic toxicity; OS trending positive but immature
Bardia et al. [25]	Randomized, open-label phase III (final analysis); SG vs TPC; prespecified secondary endpoints; post hoc by Trop-2 and HER2	267 / 262	Level I	Female ~99–100%; median age 54 vs 53; ECOG 0/1 45%/55% vs 41%/59%; median 4 prior lines; prior ICI ~30%; brain mets allowed (≤15%); HER2-evaluable 78% (71% IHC0; 29% HER2-low); Trop-2-evaluable 60% (mostly medium/high)	SG 10 mg/kg IV D1 & D8 q21d vs single-agent TPC	Median FU 11.2 mo (SG) vs 6.3 mo (TPC)	Secondary (ITT): PFS, OS, ORR, DoR, time-to-response, safety; post hoc by Trop-2/HER2	PFS: 4.8 vs 1.7 mo, HR 0.41; OS: 11.8 vs 6.9 mo, HR 0.51; benefit across Trop-2 quartiles and HER2 strata	Manageable safety; ≤5% TRAE discontinuations; no treatment-related deaths; frequent ≥G3: neutropenia, diarrhea, leukopenia	Final analysis confirms SG superiority for PFS/OS across Trop-2/HER2; manageable safety; supports SOC in ≥2L mTNBC
Diéras et al. [26]	Randomized, open-label, international phase III RCT; T-DM1 vs capecitabine+lapatinib; coprimary PFS (independent) & OS	495 / 496 (safety 490 / 488)	Level I	Median age 53; ECOG 0/1 ~60–63% / 35–39%; visceral 67–68%; HR+ 53–57%; balanced regions	T-DM1 3.6 mg/kg IV q3w vs Capecitabine 1000 mg/m² BID D1–14 q3w + Lapatinib 1250 mg daily; protocol dose mods; independent PFS review	Final OS DCO Dec 31, 2014; median FU 47.8 vs 41.9 mo	Coprimary: PFS (independent), OS; Safety: CTCAE v3.0	OS: 29.9 vs 25.9 mo, HR 0.75; sensitivity (censor crossover) 29.9 vs 24.6, HR 0.69. Prior PFS 9.6 vs 6.4 mo, HR 0.65	≥G3 AEs: 48% vs 60%. XL: diarrhea 21%, hand-foot 18%, vomiting 5%. T-DM1: thrombocytopenia 14%, AST↑ 5%, anemia 4%. AE-related deaths 3 vs 2	T-DM1 improved OS despite crossover, with fewer high-grade AEs; supports post-trastuzumab/taxane use
Krop et al. [27]	Randomized, open-label, multicenter phase III RCT; T-DM1 vs TPC; coprimary OS & investigator-assessed PFS; crossover allowed	404 / 198 (safety 403 / 184)	Level I	Median age 53–54; ECOG 0/1/2 ≈ 45%/50%/5% vs 41%/51%/8%; visceral 75–76%; HR+ ~52%; brain mets 10–14%	T-DM1 3.6 mg/kg IV q3w vs TPC per practice (single-agent chemo/hormonal/HER2-directed ± combos); scans q6w then q12w	DCO Feb 13, 2015; median FU 30.5 mo; 47% crossed to T-DM1	Coprimary: OS, PFS; Secondary: ORR, DoR, 6/12-mo survival, safety; QoL collected	OS: 22.7 vs 15.8 mo, HR 0.68 (p=0.0007); sensitivity (censor crossover) HR 0.58 (p=0.0002). PFS: 6.2 vs 3.3 mo, HR 0.53	≥G3 AEs: 40% vs 47%; higher neutropenia/febrile neutropenia/diarrhea in TPC; higher thrombocytopenia/hemorrhage in T-DM1; AE-related deaths 9 vs 3	T-DM1 significantly improved OS despite crossover; toxicity profiles distinct and manageable
Modi et al. [28]	Randomized, open-label phase III RCT; T-DXd vs physician’s-choice chemo; primary PFS (BICR) in HR+ cohort; key secondary PFS (all) & OS	373 / 184 (overall ITT); 331 / 163 (HR+ cohort)	Level I	Median age 57.5 vs 55.9; ~100% female; regions EU/Israel ~45%, Asia ~39%, NA ~16–18%; HER2-low: IHC1+ ~58%, IHC2+/ISH- ~42%; ECOG 0/1 ~54/46%; visceral common; brain mets 6.4% vs 4.3%; median 3 prior lines; prior CDK4/6 in HR+ ~70%	T-DXd 5.4 mg/kg IV q3w vs eribulin/capecitabine/nab-paclitaxel/paclitaxel/gemcitabine; BICR; ILD adjudication/management	Primary DCO Jan 11, 2022; median survival FU 18.4 mo	Primary: PFS (BICR, HR+). Key secondary: PFS (all), OS (HR+ & all); others: inv. PFS, ORR, DoR, safety	HR+: PFS 10.1 vs 5.4 mo, HR 0.51; OS 23.9 vs 17.5 mo, HR 0.64. All: PFS 9.9 vs 5.1, HR 0.50; OS 23.4 vs 16.8, HR 0.64. ORR (all): 52.3% vs 16.3%; DoR 10.7 vs 6.8	≥G3 AEs: 52.6% vs 67.4%. Any-grade T-DXd: nausea 73%, fatigue 47.7%, alopecia 37.7%; ≥G3: neutropenia 13.7%, anemia 8.1%, fatigue 7.5%. ILD/pneumonitis: 12.1% total (G1–2 10.0%; G3 1.3%; G5 0.8%); median onset ~129 days; discontinuation 16.2% vs 8.1%	T-DXd improved PFS/OS/ORR across HR subgroups; ILD requires vigilance
Perez et al. [29]	Randomized, three-arm, international phase III RCT; T-DM1 vs T-DM1+pertuzumab vs trastuzumab+taxane; primary PFS (independent)	T-DM1 367 / T-DM1+P 363 / H+taxane 365	Level I	HER2+ advanced/MBC; no prior therapy for advanced disease; baseline balanced (ECOG 0 ~56–58%, ECOG 1 ~42–43%; ER/PR+ ~54–57%; visceral ~66–70%; measurable ~88–90%)	T-DM1 3.6 mg/kg IV q3w ± pertuzumab vs trastuzumab + docetaxel/paclitaxel; RECIST v1.1 independent review	Median FU ≈ 35 mo	Primary: PFS (independent). Secondary: OS, ORR, DoR, HRQoL, safety	PFS (mo): 13.7 (H+taxane) vs 14.1 (T-DM1) vs 15.2 (T-DM1+P); both T-DM1 arms non-inferior, neither superior; ORR: 67.9% vs 59.7% vs 64.2%; DoR: 12.5 vs 20.7 vs 21.2	≥G3 AEs: 54.1% (H+taxane) vs 45.4% (T-DM1) vs 46.2% (T-DM1+P); notable: neutropenia/febrile neutropenia/diarrhea higher in control; thrombocytopenia/AST↑/anemia higher in T-DM1 arms; LVEF decline <50% with ≥15-pt drop: 0.8% vs 4.5% vs 2.5%	T-DM1±P achieved non-inferior PFS with lower high-grade toxicity and better HRQoL durability; no PFS superiority over control
Rugo et al. [30]	Randomized, open-label, global phase III RCT; SG vs physician’s-choice chemo; primary PFS (BICR), key secondary OS	272 / 271 (safety 268 / 249)	Level I	Median age 56; ~99% female; visceral mets 95%; prior CDK4/6 ~99%; median 3 prior metastatic chemo lines; ECOG 0/1 ≈ 43%/57%	SG 10 mg/kg IV D1 & D8 q21d vs eribulin/vinorelbine/capecitabine/gemcitabine; stratified by prior lines, visceral disease, endocrine-therapy duration; scans q6w then q12w	DCO Jan 3, 2022; median FU ~10.2 mo (11.3 SG; 9.8 chemo)	Primary: PFS (BICR). Key secondary: OS (hierarchical). Others: ORR, CBR, DoR, PROs, safety	PFS: 5.5 vs 4.0 mo, HR 0.66 (P=0.0003); 6-mo 46% vs 30%, 12-mo 21% vs 7%. OS (interim): 13.9 vs 12.3 mo, HR 0.84 (P=0.14). ORR 21% vs 14%; CBR 34% vs 22%; DoR 7.4 vs 5.6 mo; PROs favored SG (global health HR 0.74; fatigue HR 0.76)	Any-grade (SG vs chemo): neutropenia 70% vs 54%, diarrhea 57% vs 16%, nausea 55% vs 31%, alopecia 46% vs 16%; ≥G3: neutropenia 51% vs 38%, leukopenia 9% vs 5%, diarrhea 9% vs 1%, anemia 6% vs 3%; FN 5% vs 4%; discontinuations 6% vs 4%; deaths 6 vs 0 (one SG-related); ILD/pneumonitis 0% with SG (1% chemo)	SG significantly improved PFS with manageable toxicity; OS favored SG but not significant at interim; QoL generally better

Quality Assessment of Included Studies

The Cochrane Risk of Bias 2.0 (RoB 2) tool (The Cochrane Collaboration, London, UK) was applied to assess methodological quality across the included randomized controlled trials. Five domains formed the evaluation framework: (1) randomization process, (2) deviations from intended interventions (effect of assignment), (3) missing outcome data, (4) measurement of outcomes, and (5) selection of the reported results. Each investigation received domain-specific classifications-Low risk, Some concerns, or High risk. These individual assessments contributed to an overall risk judgment for each trial.

**Figure 2:**
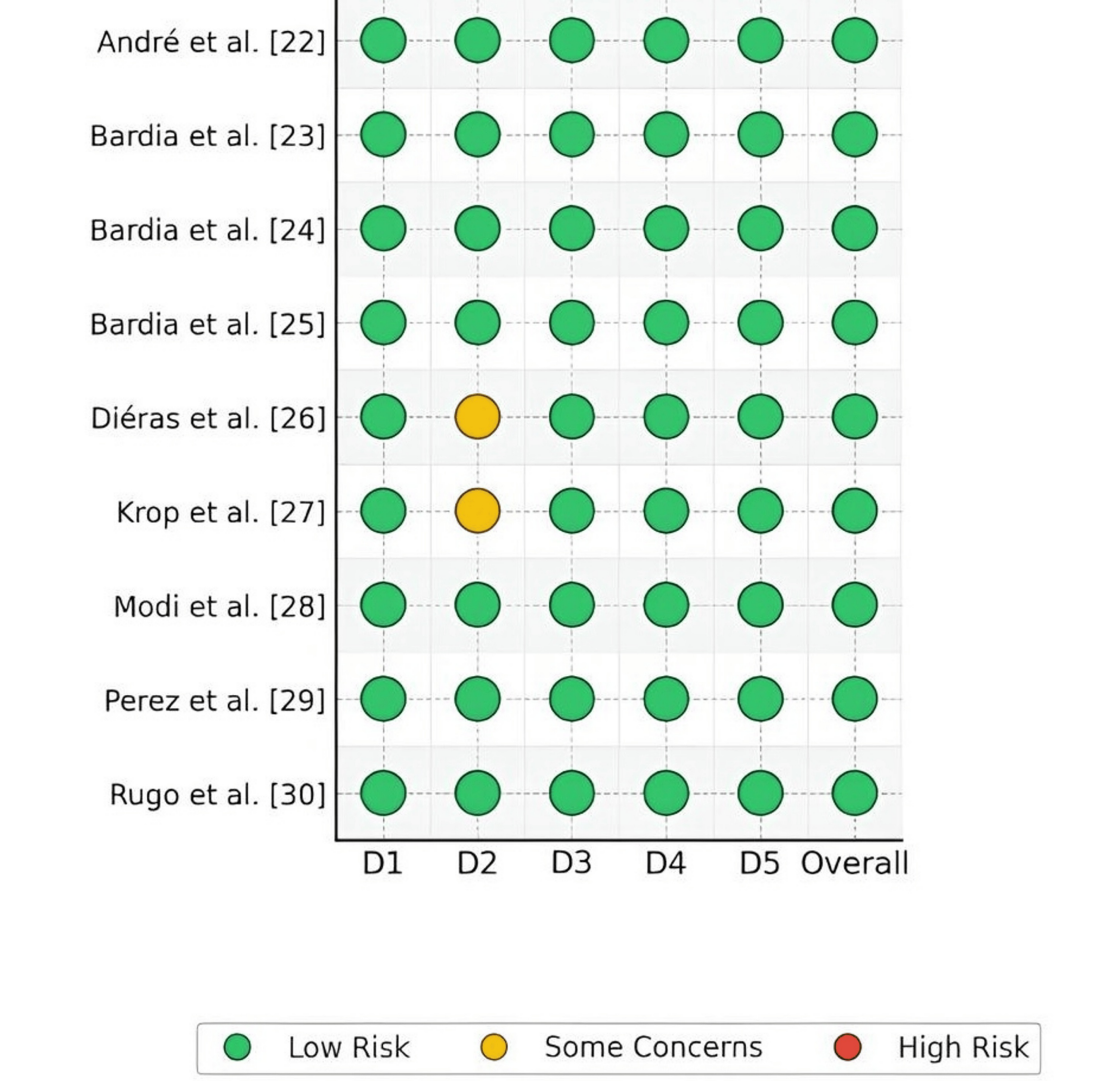
Systematic evaluation of potential bias among incorporated studies employing the Cochrane RoB2 instrument D1: Randomization Process | D2: Deviations from Intended Interventions | D3: Missing Outcome Data | D4: Measurement of Outcomes | D5: Selection of Reported Results

Across the nine phase III trials, most investigations exhibited low risk of bias in all domains, reflecting robust methodology (central/randomized allocation, prespecified analyses, BICR for PFS, and objective OS). A few trials warranted some concerns in specific domains-primarily related to crossover from control to experimental therapy (which can dilute OS effects under the “deviations from intended interventions” domain) or long follow-up with attrition (missing outcome data). No study was judged high risk overall. Table 2 and Figure 2 provide RoB 2 evaluations.

**Table 2:** Quality assessment for included studies using the Cochrane Risk of Bias Tool 2.0 (RoB2)

Study (Author, Ref)	Randomization Process	Deviations from Intended Interventions	Missing Outcome Data	Measurement of Outcomes	Selection of Reported Results	Overall Risk of Bias
André et al. [22]	Low risk	Low risk	Low risk	Low risk	Low risk	Low risk
Bardia et al. [23]	Low risk	Low risk	Low risk	Low risk	Low risk	Low risk
Bardia et al. [24]	Low risk	Low risk	Low risk	Low risk	Low risk	Low risk
Bardia et al. [25]	Low risk	Low risk	Low risk	Low risk	Low risk	Low risk
Diéras et al. [26]	Low risk	Some concerns (crossover from control to T-DM1 may bias OS toward null)	Low risk	Low risk	Low risk	Low risk
Krop et al. [27]	Low risk	Some concerns (post-randomization crossover to T-DM1 may affect OS)	Low risk	Low risk	Low risk	Low risk
Modi et al. [28]	Low risk	Low risk	Low risk	Low risk	Low risk	Low risk
Perez et al. [29]	Low risk	Low risk	Low risk	Low risk	Low risk	Low risk
Rugo et al. [30]	Low risk	Low risk	Low risk	Low risk	Low risk	Low risk

Results of the Meta-Analysis

**Figure 3:**
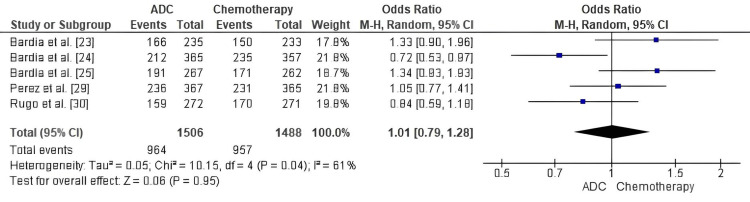
Forest plot comparing antibody–drug conjugates (ADCs) and standard chemotherapy for progression-free survival (PFS) ADC: Antibody–Drug Conjugate; OR: Odds Ratio; CI: Confidence Interval; PFS: Progression-Free Survival. Source: [23-30]

Comparison of ADC and standard chemotherapy for PFS: Forest-plot meta-analysis demonstrated no statistically significant difference in PFS between ADCs and standard chemotherapy (pooled OR = 1.01; 95% CI: 0.79-1.28; p = 0.95). Moderate heterogeneity (I² = 61%) reflected variation in study populations and treatment regimens, yet the overall direction of effect indicated comparable disease control between both groups (Figure 3).

**Figure 4:**
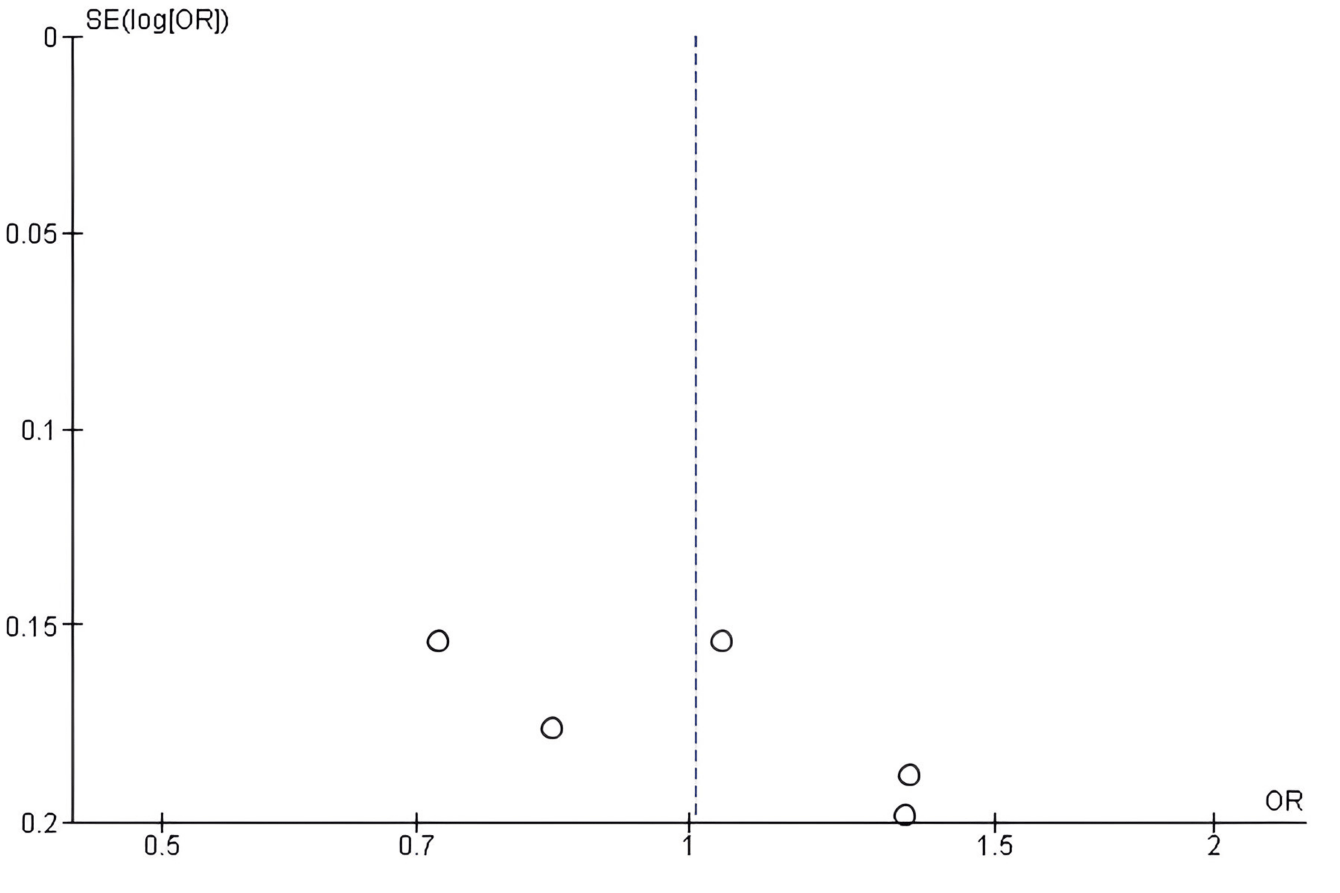
Funnel plot assessing publication bias for studies comparing antibody–drug conjugates (ADCs) and standard chemotherapy with respect to progression-free survival (PFS) ADC: Antibody–Drug Conjugate; SE: Standard Error; OR: Odds Ratio; PFS: Progression-Free Survival

Publication bias assessment for PFS: No evidence of significant publication bias was found when the PFS results were analyzed using a funnel plot. The results of the funnel-plot analysis are largely symmetrical (Figure 4), with some asymmetry that may be the result of sample size variations and inter-study heterogeneity rather than selective reporting. The conclusion that the distribution pattern is due to chance rather than bias is supported by the non-significant Egger's regression test results (p > 0.05).

**Figure 5:**
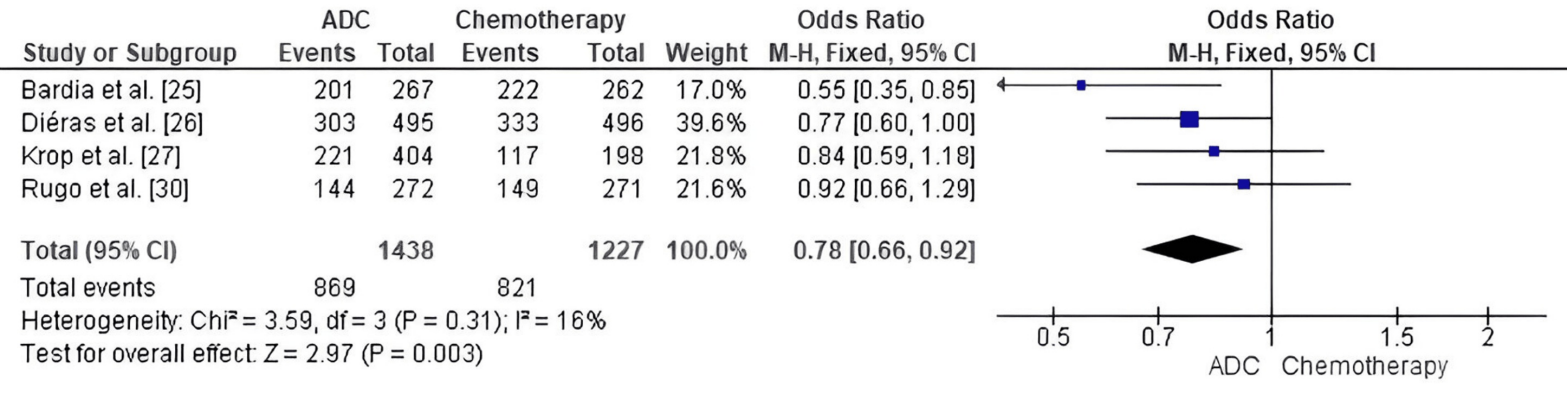
Forest plot comparing antibody–drug conjugates (ADCs) and standard chemotherapy for overall survival (OS) ADC: Antibody–Drug Conjugate; OR: Odds Ratio; CI: Confidence Interval; OS: Overall Survival. Source: [25-30]

Comparison of ADC and standard chemotherapy for OS: A forest-plot analysis of ADCs versus standard chemotherapy in terms of OS for patients with MBC showed a pooled odds ratio (OR) of 0.78 (95% CI: 0.66-0.92) in favor of ADCs, with a statistically significant survival benefit (p = 0.003) and low heterogeneity (I² = 16%, p = 0.31) across studies for ADC type, patient subgroups, and prior treatment exposure, all demonstrating strong evidence that ADCs confer a survival advantage compared with standard chemotherapy (Figure 5).

**Figure 6:**
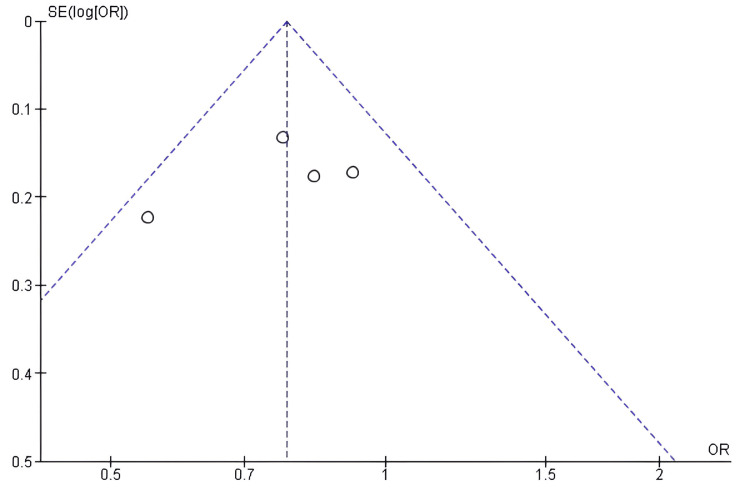
Funnel plot assessing publication bias for studies comparing antibody–drug conjugates (ADCs) and standard chemotherapy with respect to overall survival (OS). ADC: Antibody–Drug Conjugate; SE: Standard Error; OR: Odds Ratio; OS: Overall Survival.

Publication bias assessment for OS: Funnel-plot analysis for overall survival demonstrated symmetrical study distribution around the central effect line, indicating the absence of significant publication bias (Figure 6). Minor asymmetry appeared upon visual examination. This pattern reflected sampling variation in smaller trials rather than systematic bias. Egger's regression test yielded non-significant results (p > 0.05).

**Figure 7:**
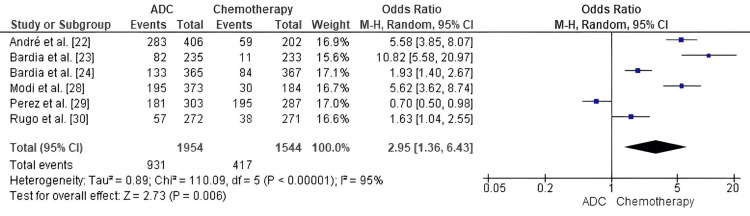
Forest plot comparing antibody–drug conjugates (ADCs) and standard chemotherapy for objective response rate (ORR) ADC: Antibody–Drug Conjugate; OR: Odds Ratio; CI: Confidence Interval; ORR: Objective Response Rate. Source: [22-30]

Comparison of ADC and standard chemotherapy for ORR: Forest-plot analysis of the ORR in patients with MBC treated with ADCs and standard chemotherapy found a pooled OR of 2.95 (95% CI: 1.36-6.43) showing significant benefit of ADC therapy over standard chemotherapy with low heterogeneity across studies (p = 0.006; I² = 95%, p < 0.00001), which may be due to differences in ADC types, prior treatment lines, and assessment criteria (RECIST versions); however, results consistently favored ADC treatment, which suggests that ADCs have higher response rates compared with conventional chemotherapy (Figure 7).

**Figure 8:**
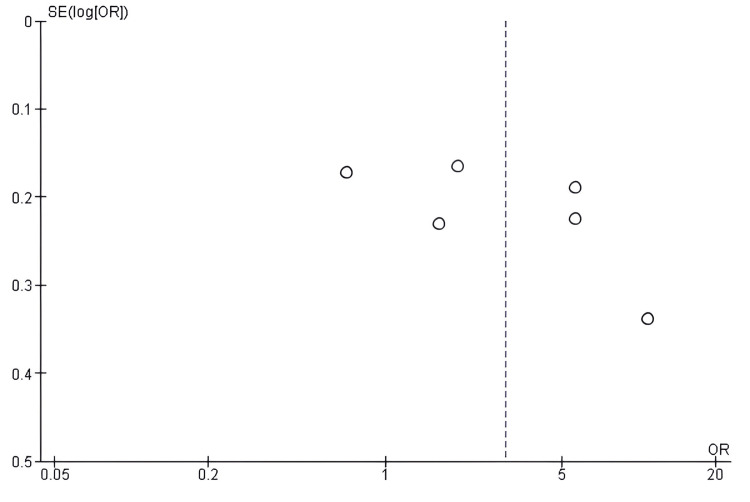
Funnel plot assessing publication bias for studies comparing antibody–drug conjugates (ADCs) and standard chemotherapy with respect to the objective response rate (ORR) ADC: Antibody–Drug Conjugate; SE: Standard Error; OR: Odds Ratio; ORR: Objective Response Rate.

Publication bias assessment for ORR: Evaluation of publication bias through funnel-plot analysis for ORR outcomes revealed minor asymmetry in the distribution, potentially attributable to inter-trial heterogeneity, wherein studies with smaller sample sizes demonstrated more pronounced treatment effects. However, the non-significant result of Egger's test (p > 0.05) suggested that this asymmetry stemmed from variations in sample size and clinical heterogeneity rather than from selective reporting practices (Figure 8).

Comparison of ADC and standard chemotherapy for Grade ≥ 3 AEs: The frequency of Grade ≥ 3 AEs in MBC patients was evaluated through forest-plot methodology, contrasting outcomes between ADCs and standard chemotherapy. The analysis demonstrated a pooled OR of 0.68 (95% CI: 0.44-1.04), which reflected a statistically non-significant trend (p = 0.08) favoring ADC therapy with respect to severe AEs.

**Figure 9:**
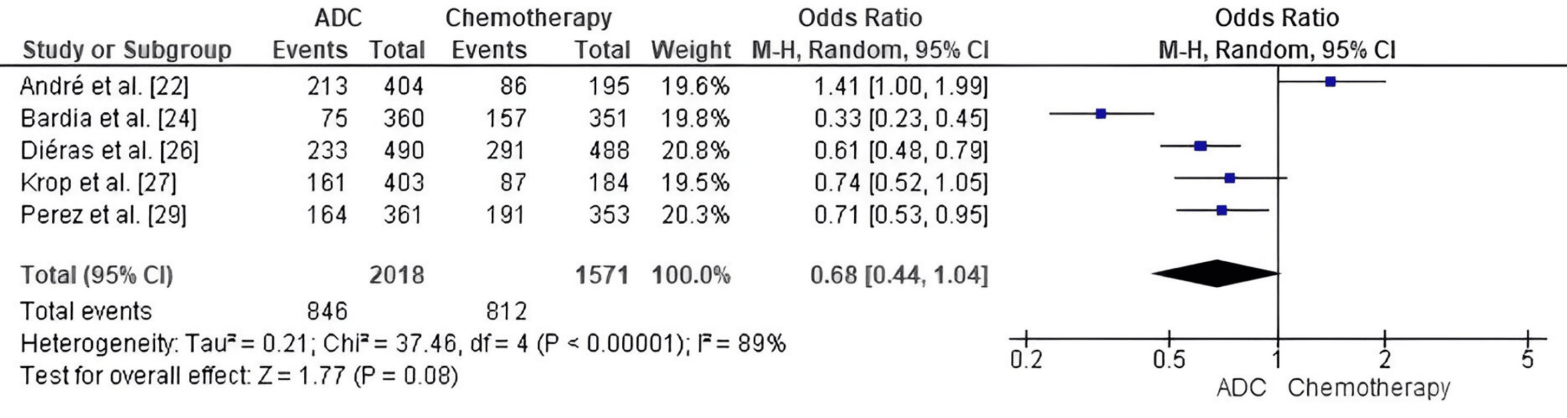
Forest plot comparing antibody–drug conjugates (ADCs) and standard chemotherapy for Grade ≥ 3 adverse events ADC: Antibody–Drug Conjugate; OR: Odds Ratio; CI: Confidence Interval; AE: Adverse Events. Source: [22-29]

There was substantial heterogeneity among studies (I² = 89%, p < 0.00001), but there was a strong direction of effect in favor of ADCs, indicating that, on average, the risk of high-grade toxicity for ADCs may be lower than that for traditional chemotherapy (Figure 9).

**Figure 10:**
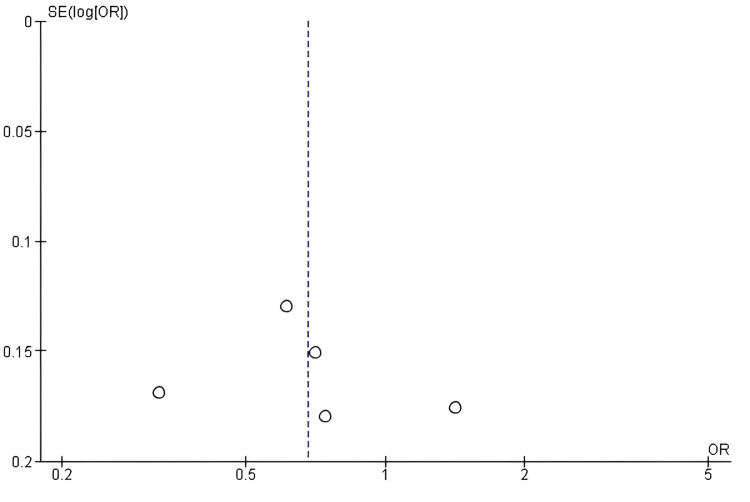
Funnel plot assessing publication bias for studies comparing antibody–drug conjugates (ADCs) and standard chemotherapy with respect to Grade ≥ 3 adverse events ADC: Antibody–Drug Conjugate; SE: Standard Error; OR: Odds Ratio; AE: Adverse Events.

Publication bias assessment for Grade ≥ 3 AEs*: *The funnel-plot analysis for Grade ≥ 3 AEs showed no apparent evidence of publication bias, as the studies were symmetrically distributed around the central line of effect and there was no asymmetry around the pooled estimate in the funnel plot (Figure 10); Egger’s test was not significant (p > 0.05), further validating the reliability of the combined analysis.

Discussion

A meta-analysis of ADCs versus standard chemotherapy in MBC was performed, which summarized high-level evidence demonstrating that ADCs deliver clinically relevant benefits in survival and tumor response with no compromise in safety. In nine phase III randomized trials (6,186 patients), ADCs showed a statistically significant improvement in OS and ORR, comparable PFS, and no significant increase in grade ≥3 AEs.

While pooled analysis failed to show a statistically significant PFS advantage for ADCs (OR = 1.01, p = 0.95), individual studies showed heterogeneity of benefit based on agent class and patient population: André et al. [22] reported a significant PFS benefit with trastuzumab deruxtecan (T-DXd) vs physician’s-choice chemotherapy (17.8 vs 6.9 months), whereas Bardia et al. [23] found that SG improved PFS from 1.7 to 5.6 months in heavily pretreated triple-negative disease, and Bardia et al. [24] showed a 37% lower risk of progression for datopotamab deruxtecan (Dato-DXd) vs chemotherapy. Conversely, Perez et al. [29] and Diéras et al. [26] reported only modest or non-significant differences when comparing T-DM1 with trastuzumab-taxane or lapatinib-capecitabine combinations, which may represent the cumulative effect of prior exposure to HER2-directed therapies. While the magnitude of benefit varied, ADCs numerically favored disease control in almost all trials, underscoring their continued activity in refractory settings.

This improvement in OS is one of the most important distinguishing advantages of ADCs compared to chemotherapy. OS benefit was significant, and there was low heterogeneity among the trials (OR = 0.78, p = 0.003); in the DESTINY-Breast02 trial, median OS was 39.2 months with T-DXd versus 26.5 months in the comparator group [22]. Similarly, SG achieved nearly twofold greater OS (12.1 months compared to 6.7 months) among patients with metastatic triple-negative breast cancer [23,25], whereas Dato-DXd exhibited encouraging preliminary OS outcomes in hormone receptor-positive disease [24]. Furthermore, Modi et al. [28] established that T-DXd conferred substantial OS gains in HER2-low patients (23.9 versus 17.5 months; HR 0.64), a population that conventional HER2-targeted therapies had not previously been deemed effective for treating. Previous trials of T-DM1 by Diéras et al. [26] and Krop et al. [27] showed longer survival compared with chemotherapy, even with crossover, further establishing ADCs as life-prolonging agents in HER2-positive, HER2-low, and triple-negative populations.

ADCs have demonstrated better cytotoxic delivery and tumor selectivity than other chemotherapeutics, which likely explains the significantly higher objective response rate with ADC therapy compared to chemotherapy (OR = 2.95, p = 0.006). T-DXd resulted in 70% versus 29% ORR compared with control (André et al. [22]), and 52.3% versus 16.3% in HER2-low cohorts (Modi et al. [28]). In triple-negative disease, Bardia et al. [23,25] reported ORRs of 35% versus 5% with SG, and 36.4% versus 22.9% with Dato-DXd versus chemotherapy [24], which highlights the ability of ADCs to cause more profound and sustained tumor regression. The MARIANNE trial (Perez et al. [29]) was the only one that showed equivalence between T-DM1-based and taxane-based regimens, possibly reflecting the incremental nature of first-generation ADCs in comparison with more modern agents.

In terms of safety, ADCs showed a trend toward fewer grade ≥3 Aes (OR = 0.68, p = 0.08), supporting their improved tolerability compared with conventional chemotherapy, and Diéras et al. [26] reported lower incidence of severe toxicity with T-DM1 (48% vs 60%), with fewer hematologic and gastrointestinal Aes versus control regimens [27]. Dato-DXd led to significantly fewer high-grade hematologic toxicities (20.8% vs 44.7%) [24], and SG exhibited expected neutropenia and diarrhea that could be well controlled with supportive care [23,25]. T-DXd has a significant risk of ILD [22,28], but most cases were low grade and reversible with prompt identification and corticosteroid treatment. ADCs offer a different toxicity profile, less nonspecific myelosuppression and mucositis but more agent-specific toxicities, which enable prolonged treatment while maintaining quality of life.

Limitations

The limitations of this meta-analysis include inter-study heterogeneity, variations in patient populations and prior treatments, and crossover from control to ADC arms, which may have diluted overall survival effects. The lack of uniformity in definitions and reporting of biomarkers also limited more detailed subgroup analysis.

## Conclusions

The high selectivity afforded by targeted antibodies combined with potent cytotoxic effects has led to improved OS and higher response rates than standard chemotherapy in MBC, without a significant increase in severe toxicity (confirmed that ADCs are a mainstay of modern breast cancer therapy), although continued vigilance for drug-specific toxicities, including interstitial lung disease associated with trastuzumab deruxtecan, is warranted. The overall benefit-risk balance favors the use of these novel agents as part of ADC-based regimens; further research will concentrate on biomarker-driven approaches to improve patient selection and combination strategies in order to extend survival and quality of life for patients with advanced disease.
